# Clinical significance of monitoring *ESR1* mutations in circulating cell-free DNA in estrogen receptor positive breast cancer patients

**DOI:** 10.18632/oncotarget.8839

**Published:** 2016-04-19

**Authors:** Takashi Takeshita, Yutaka Yamamoto, Mutsuko Yamamoto-Ibusuki, Toko Inao, Aiko Sueta, Saori Fujiwara, Yoko Omoto, Hirotaka Iwase

**Affiliations:** ^1^ Department of Breast and Endocrine Surgery, Graduate School of Medical Science, Kumamoto University, Chuo-ku, Kumamoto, 860–8556, Japan; ^2^ Department of Molecular-Targeting Therapy for Breast Cancer, Kumamoto University Hospital, Chuo-ku, Kumamoto, 860–8556, Japan; ^3^ Department of Endocrine and Breast Surgery, Graduate School of Medical Science, Kyoto Prefectural University of Medicine, Hirokoji Agaru, Kawaramachi-dori, Kamigyo-ku, Kyoto, 602–0841, Japan

**Keywords:** estrogen receptor-positive metastatic breast cancer, acquired endocrine therapy resistance, cell-free DNA ESR1 mutations in plasma, droplet digital PCR

## Abstract

**Background:**

The measurement of circulating cell-free DNA (cfDNA) may transform the management of breast cancer patients. We aimed to investigate the clinical significance of sequential measurements of *ESR1* mutations in primary breast cancer (PBC) and metastatic breast cancer (MBC) patients.

**Results:**

*ESR1* mutations ratio in the PBC groups was used as the minimum cutoff for determining increases in cfDNA *ESR1* mutation ratio. An increase in cfDNA *ESR1* mutations was found in 13 samples of cfDNA from 12 (28.6%) out of 42 MBC patients. A total of 10 (83.3%) out of 12 MBC patients with increase cfDNA *ESR1* mutations showed a poor response to treatment. In survival analysis, increase cfDNA *ESR1* mutations may predict a shorter duration of post-endocrine-therapy effectiveness (*P* = 0.0033).

**Methods:**

A total of 119 patients (253 plasma samples) with breast carcinoma were enrolled in this study. Cases were selected if archival plasma samples were available from PBC before and after treatment and from MBC gathered more than twice at the time of progression. cfDNA was isolated from the 77 PBC patients (154 plasma samples) and from the 42 MBC patients (99 plasma samples). To investigate any changes in each cfDNA *ESR1* mutation before and after treatment, we analyzed the difference with cfDNA *ESR1* mutations ratio in the first blood sample using droplet digital polymerase chain reaction (ddPCR).

**Conclusions:**

We demonstrate that ddPCR monitoring of the recurrent *ESR1* mutation in cfDNA of MBC patients is a feasible and useful method of providing relevant predictive information.

## INTRODUCTION

In hormone receptor (HR)-positive metastatic breast cancer (MBC) without life-threating visceral metastases, aromatase inhibitors (AIs) or tamoxifen are the first-line treatments of choice because of their effectiveness balanced against their side effects [1]. However, all initially hormone-dependent breast cancers acquire anti-estrogen resistance after repeated endocrine therapies and, eventually, become hormone-independent [2]. Recently, *ESR1*-activating mutations have been postulated as the key potential mechanisms underlying the failure of endocrine therapies. *ESR1* mutations were first identified in patient xenograft studies reported almost two decades ago [3, 4] and next generation sequencing (NGS) studies revealed that *ESR1* point mutations in a hot spot confined to Tyr537 and Asp538 act as a driver of endocrine therapy resistance [5–8]. In particular, the representative four *ESR1* ligand binding domain (LBD) “hot spot” mutations, *ESR1* Y537S, Y537N, Y537C, and D538G, which cover more than 80% of *ESR1* mutations associated with acquired resistance to antiestrogen therapy [5–7]. The detection of these mutations may be useful as biomarker of resistance to endocrine therapy and could help in choosing the most appropriate treatment for HR+ MBC [8–11]. In order to use *ESR1* LBD mutations as biomarker for disease monitoring, the *ESR1* genotyping should be performed whenever a disease progresses. However, when that is monitored by the tumor tissues, there are the following three limitations. Firstly, tumor biopsies are inconvenient from a scheduling perspective, especially when the tumor site is not easily accessible. Secondly, the method of sample preservation (e.g. formalin-fixed paraffin-embedded) and intratumoral or intertumoral heterogeneity, also hamper the use of tumor tissue material. Finally, taking biopsies from tumor tissue always carries the risk of clinical complications [12].

Circulating cell-free DNA (cfDNA) analysis has been developed as a way of overcoming these limitations and providing relevant predictive information related to the tumor tissue [13–18]. In order for detection of cfDNA *ESR1* mutations to prove clinically useful, *ESR1* rare point mutations must be detected from small quantities of short lengths of DNA in plasma [19]. Droplet digital polymerase chain reaction (ddPCR) technology could solve this problem by means of its superior accuracy [20]. Technically, we had already evaluated the quantitative performance of ddPCR using four representative *ESR1* LBD mutant molecules, *ESR1* Y537S, Y537N, Y537C, and D538G in breast tumor tissue [21]. Additionally, using this assay, we could detect a small amount of cfDNA *PIK3CA* major mutations in early-stage triple negative breast cancer [22].

In this retrospective study, we used ddPCR to investigate the clinical significance of tracking four representative types of *ESR1* LBD mutations in 253 plasma samples from 119 breast cancer patients, of which 99 were from 42 MBC patients and 154 were from 77 advanced ER-positive primary breast cancer (PBC) patients. To our knowledge, this is a leading study to evaluate the clinical significance of sequential measurements of *ESR1* mutations in a large series of plasma samples from patients with PBC and MBC.

## RESULTS

### Patient characteristics

**Figure 1:**
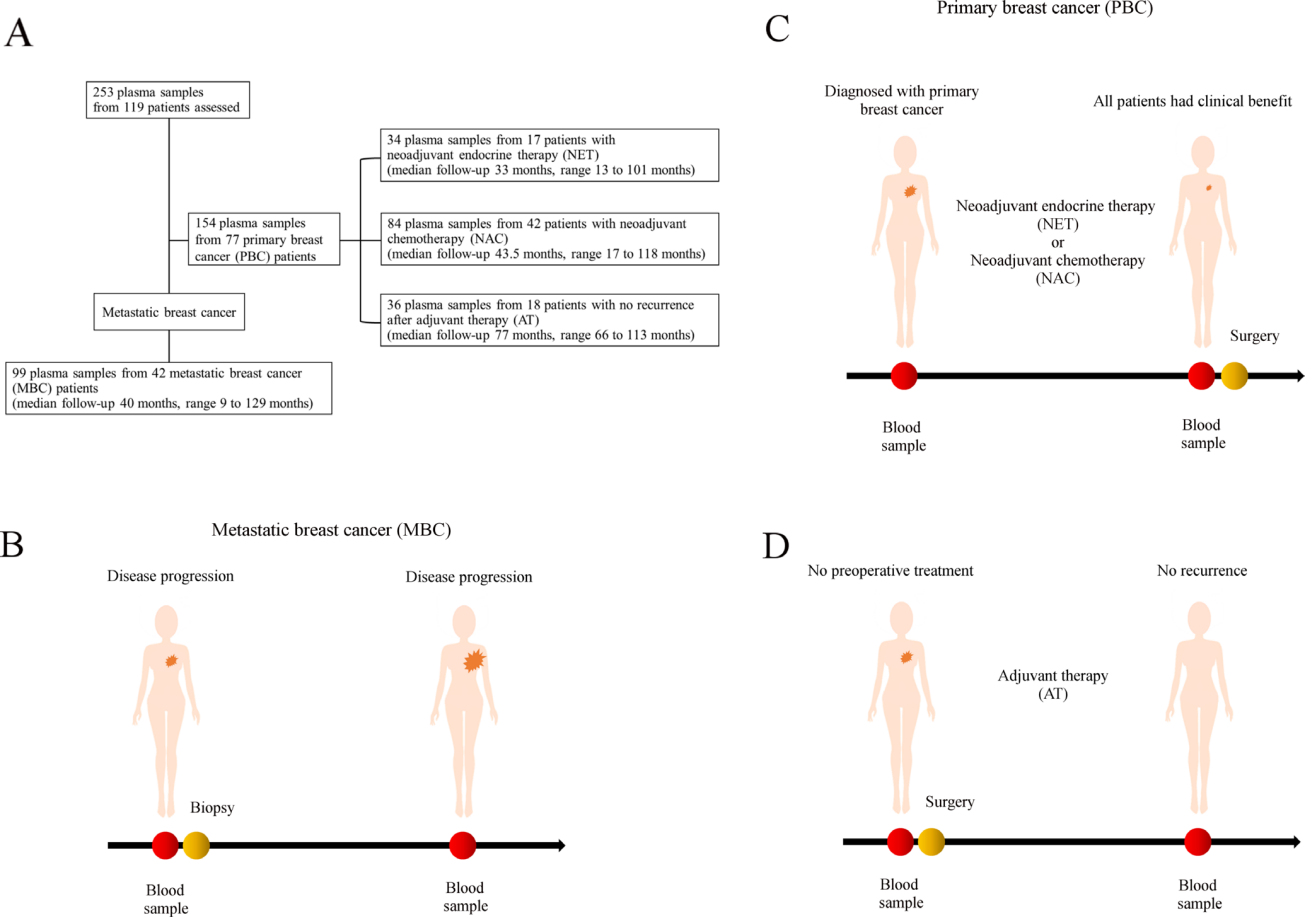
(**A**) Schematic representation of the study protocol (**B–D**). Study schema. For 119 women with breast cancer, personalized droplet digital PCR assays were used to quantify *ESR1* DNA sequences in the circulating cell-free DNA (cfDNA) isolated from 253 patient blood plasma samples taken serially during the clinical course. cfDNA was isolated from 154 plasma samples from the 77 primary breast cancer (PBC) patients and (B) 99 plasma samples from the 42 metastatic breast cancer (MBC) patients. In 77 PBC patients, (C) 17 patients were treated by neoadjuvant endocrine therapy (NET), (C) 42 patients were treated by neoadjuvant chemotherapy (NAC), and (D) 18 patients were treated by adjuvant therapy only (AT). We analyzed how cfDNA *ESR1* mutations change following endocrine therapy or chemotherapy by comparing the NET group with the NAC group. We also investigated how cfDNA *ESR1* mutations change following tumor resection by comparing the AT group with the other PBC groups.

A total of 119 patients (253 plasma samples) with breast carcinoma were enrolled in this study. A total of 77 women with PBC and 42 with MBC were evaluated. Of the 77 PBC patients, 17 were treated by neoadjuvant endocrine therapy (NET), 42 were treated by neoadjuvant chemotherapy (NAC), and 18 were not treated before surgery, but were treated by adjuvant therapy (AT) (Figure 1). To investigate changes in cfDNA *ESR1* mutations following endocrine therapy or chemotherapy and adjuvant treatment for more than 5 years, we created subgroups of patients who treated by NET, NAC, and AT in the PBC group.

The patient demographics and baseline characteristics of PBC and MBC are presented in Table 1. The median age of the patients at first blood draw was 67 years (range, 41–82) in the NET group, 50 years (range, 31–70) in the NAC group, 62 years (range, 37–76) in the AT group, and 58 years (range, 31–82) in the MBC group. In the PBC groups, NAC patients had a higher stage, higher histological grade, lower ERα immunostaining, and positive Ki67 labeling index (LI). Of the primary clinical stage MBC patients, 16 patients (38.1%) were categorized as stage IV and a total of 14 out of 42 cases (33.3%) were not treated before first blood sampling. A total of 2 out of 42 MBC cases were not treated until second blood draw because one had a past history of cerebral infarction and the other had microinvasive disease. The median duration of follow-up was 33 months (range, 13–101 months) in the NET group, 43.5 months (range, 17–118 months) in the NAC group, 77 months (range, 66–113 months) in the AT group, and 40 months (range, 9–129 months) in the MBC group. There was no recurrence during the observation period in any of the PBC patients.

**Table 1:** Patient characteristics

Variables	Total	No. of samples (%)			MBC
		Primary breast cancer			
		NET	NAC	AT	
	(*N* = 119)	(*N* = 17)	(*N* = 42)	(*N* = 18)	(*N* = 42)
**Age at biopsy**					
Median (range)	58 (31–82)	67 (41–82)	50 (31–70)	62 (37–76)	58 (31–82)
**Primary clinical Stage**					
I	26 (21.8)	4 (23.5)	1 (2.4)	14 (77.8)	7 (16.7)
II	59 (49.6)	11 (64.7)	29 (69)	4 (22.2)	15 (35.7)
III	17 (14.3)	2 (11.8)	12 (28.6)	0	4 (9.5)
IV	17 (14.3)	0	0	0	16 (38.1)
**Histological type**					
Invasive ductal	113 (95)	13 (76.5)	42 (100)	18 (100)	40 (95.2)
Invasive lobular	3 (2.5)	1 (5.9)	0	0	2 (4.8)
Mucinous	3 (2.5)	3 (17.6)	0	0	0
**Histological grade**					
1	45 (37.8)	5 (29.4)	10 (23.8)	12 (66.7)	18 (42.9)
2	51 (42.9)	11 (64.7)	22 (52.4)	6 (33.3)	12 (28.6)
3	20 (16.8)	0	10 (23.8)	0	10 (23.8)
Lobular	3 (2.5)	1 (5.9)	0	0	2 (4.8)
**The percentage of ERα median (25%, 75%)**	119 (100)	90 (90–95)	85 (60–90)	90 (80–91.3)	90 (70–95)
**The percentage of PgR median (25%, 75%)**	119 (100)	50 (7.5–85)	45 (5–82.5)	55 (20–90)	30 (0.75–70)
**HER2**					
Negative	16 (13.4)	16 (94.1)	34 (80.1)	17 (94.4)	36 (85.7)
Positive	103 (86.6)	1 (5.9)	8 (19.1)	1 (5.6)	6 (14.3)
**Ki67 LI**					
≤ 14	54 (45.4)	15 (88.2)	11 (26.2)	7 (38.9)	21 (50)
> 14	35 (29.4)	2 (11.8)	20 (47.6)	1 (5.5)	12 (28.6)
Unknown	30 (25.2)	0	11 (26.2)	10 (55.6)	9 (21.4)
**Therapy change: number of times before first blood sampling**					
0					14 (33.3)
1					13 (31)
2					2 (4.8)
4					3 (7.1)
5					1 (2.4)
6					2 (4.8)
8					1 (2.4)
9					3 (7.1)
10					1 (2.4)
12					2 (4.8)
**Treatment before first blood sampling**					
AI	10 (8.4)	0	0	0	10 (23.8)
SERM	4 (3.4)	0	0	0	4 (9.5)
Others	3 (2.5)	0	0	0	3 (7.1)
Chemotherapy	11 (9.2)	0	0	0	11 (26.2)
None	91 (76.5)	17 (100)	42 (100)	18 (100)	14 (33.3)
**Treatment after first blood sampling**					
AI	29 (24.4)	17 (100)	0	0	12 (28.6)
SERM	8 (6.7)	0	0	0	8 (19)
Others	6 (5)	0	0	0	6 (14.3)
Chemotherapy	56 (47.1)	0	42 (100)	0	14 (33.3)
None	20 (16.8)	0	0	18 (100)	2 (4.8)
**Treatment before second blood sampling**					
AI	29 (24.4)	17 (100)	0	0	12 (28.6)
SERM	7 (5.9)	0	0	0	7 (16.7)
Other endocrine therapy	9 (7.6)	0	0	0	9 (21.4)
Chemotherapy	54 (45.4)	0	42 (100)	0	12 (28.6)
None	20 (16.8)	0	0	18 (100)	2 (4.8)
**Treatment after second blood sampling**					
AI	55 (46.2)	16 (94.1)	15 (35.7)	10 (55.6)	14 (33.3)
SERM	29 (24.4)	0	19 (45.2)	2 (11.1)	8 (19.1)
Other endocrine therapy	10 (8.4)	0	0	0	10 (23.8)
Chemotherapy	25 (21.0)	1 (5.9)	8 (19.1)	6 (33.3)	10 (23.8)
None	0	0	0	0	0
**Treatment after third blood sampling**					
AI					0
SERM					1 (9.1)
Other endocrine therapy					4 (36.4)
Chemotherapy					6 (54.6)
None					0
**Treatment after fourth blood sampling**					
AI					0
SERM					0
Other endocrine therapy					1 (25)
Chemotherapy					3 (75)
None					0

Abbreviations: NET, neoadjuvant endocrine therapy; NAC, neoadjuvant chemotherapy; AT, adjuvant therapy; MBC, metastatic breast cancer; ERα, estrogen receptor alpha; PgR, progesterone receptor; HER2, human epidermal growth factor receptor 2; LI, labeling index; SERM, selective estrogen receptor modulator.

### Tracking cfDNA *ESR1* mutations analysis in breast cancer patients

cfDNA was isolated from 154 plasma samples from the 77 PBC patients and from 99 plasma samples from the 42 MBC patients. Plasma was collected at more than two points of the clinical course in all patients (three points in a total of 11 (26.1%) and four points in a total of 4 (9.5%) out of 42 MBC patients). As we expected, the median concentration of cfDNA was the highest in the MBC group (data not shown). In consecutive analysis of cfDNA *ESR1* mutations, we should take the following 3 things into account. Firstly, breast cancer cells with *ESR1* LBD mutations can survive and breast cancer cells with *ESR1* wild-type cannot survive by endocrine therapy [3–7, 23]. Secondly, the cfDNA extraction kit extracted DNA from plasma without distinction of double strand DNA and single strand DNA and they were parsed by one strand DNA by ddPCR. Finally, somatic mutations are present in commonly single base-pair substitutions. Therefore, we thought the allelic ratios of mutant to wild type *ESR1* were more precise biomarker than the absolute quantification or frequency of *ESR1* mutations. The ratio of mutation to wild type for the 4 *ESR1* mutations for the 1st and the 2nd blood draw in this series is shown in Figure S1. In the 1st blood draw, there was no significant difference between the PBC group and the MBC group. However, in the 2nd blood draw, cfDNA *ESR1* Y537S ratio in the MBC group was significantly higher than that in the NAC and the AT group (*P* = 0.031 and 0.038, respectively), and cfDNA *ESR1* D538G ratio was significantly higher than that in the NET group (*P* = 0.032).

**Figure 2:**
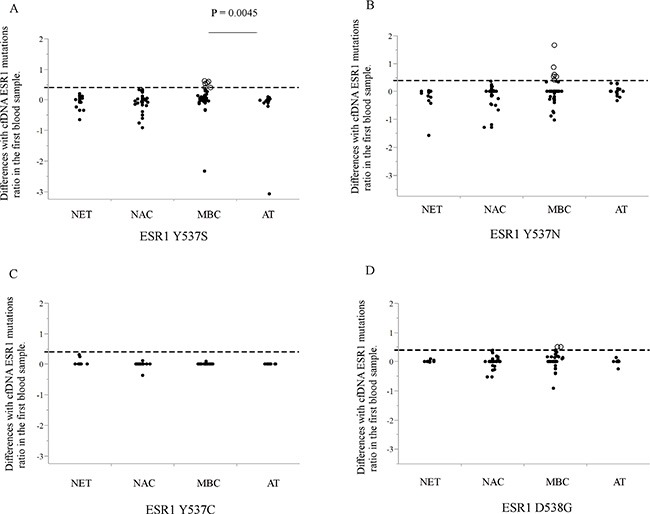
(**A–D**) The ratio of post-treatment to pre-treatment for each cfDNA *ESR1* mutation (A; *ESR1* Y537S, B; Y537N, C; Y537C, and D; D538G) are shown. All samples were measured with *ESR1* wild-type molecule and each *ESR1* mutant molecule as positive control. A water only (no template) control was run in parallel for each ddPCR reaction as negative control. *ESR1* mutations tended to be higher in the MBC group compared with other PBC groups. Whether cfDNA *ESR1* mutations ratio after treatment was increased or not, we set 0.4055 ratios gain compared with that in the first blood sample for the cut-off level of increasing cfDNA *ESR1* mutations ratio that we cannot identify in the other PBC groups (broken line shows cut-off line and open circles show cases with increasing *ESR1* mutations ratio). Using this selected cut-off point, we identified 13 increases in cfDNA *ESR1* mutations ratio during the treatment period from 12 (28.6%) out of 42 MBC patients. Abbreviations; cfDNA, cell-free DNA; ddPCR, droplet digital polymerase chain reaction; MBC, metastatic breast cancer; PBC, primary breast cancer.

**Figure 3:**
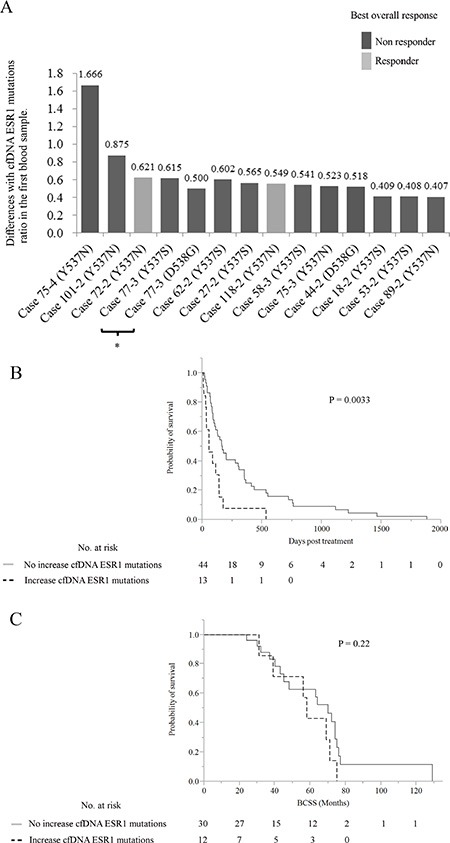
(**A**) The differences with cfDNA *ESR1* mutations ratio in the first blood sample in 13 samples with increasing cfDNA *ESR1* mutations from 12 out of 42 MBC patients are shown according to the response to treatment. Black and gray bars represent response and non-response cases, respectively. *Same sample had increasing cfDNA *ESR1* Y537S and D538G (**B, C**) Kaplan-Meier plots of the association of increases in cfDNA *ESR1* mutations with (B) TTF and (C) BCSS in 42 MBC patients. When increasing cfDNA *ESR1* mutations were defined as either positive or negative using the selected cut-off, positive cases seemed to have a shorter duration of post-treatment effectiveness than negative ones in log-lank tests (*P* = 0.0033). A total of 3 out of 12 patients with increasing cfDNA *ESR1* mutations were treated by chemotherapy. In BCSS analysis, there was no significant difference among the patients with and without increasing cfDNA *ESR1* mutations (*P* = 0.22). Abbreviations: cfDNA, cell-free DNA; MBC, metastatic breast cancer; TTF, time to treatment failure; BCSS, breast cancer-specific survival.

The paired analysis to compare pre- and post-treatment cfDNA *ESR1* mutation ratio allowed us to establish if there was a change in cfDNA *ESR1* mutations ratio in the transition from treatment-sensitive to -refractory disease for each individual patient. *ESR1* mutation tracking in cfDNA in PBC and MBC patients and the difference with each cfDNA *ESR1* mutation ratio of the first blood draw (*ESR1* Y537S, Y537N, Y537C, and D538G) are shown in Figure 2. By examining the difference with cfDNA *ESR1* mutations ratio in the first blood draw for each patient, we were able to create subgroups of patients whose cfDNA *ESR1* mutations ratio exhibited either an increase or not in all patients. All samples were compared with the *ESR1* wild-type molecule and each *ESR1* mutant molecule as positive control. A water only (no template) control was run in parallel for each ddPCR reaction as negative control. An increase in cfDNA *ESR1* Y537S ratio was statistically significant in the MBC group compared with the AT group (*P* = 0.0045), but there was no significant difference in *ESR1* Y537N, *ESR1* Y537C, and *ESR1* D538G between all PBC groups and the MBC group. *ESR1* mutations ratio tended to be increased in the MBC group compared with the other PBC groups (Figure S2). We established a cut-off value of the increase in cfDNA *ESR1* mutations based on PBC groups. Therefore, we set 0.4055 ratios gain compared with that in the first blood sample for the cut-off level of increasing cfDNA *ESR1* mutations ratio. According to the selected cut-off point, we identified 13 increases in cDNA *ESR1* mutations during treatment from 12 (28.6%) out of 42 MBC patients; detailed descriptions of these patients are given in Figure 3A and Table 2. Systemic endocrine therapy was administered, although many patients also received prior chemotherapy. Among these 12 patients, 6 patients (50%) each had increasing cfDNA *ESR1* Y537S and Y537N, and 2 patients (16.7%) had increasing cfDNA *ESR1* D538G. Interestingly, 1 patient had 2 increasing cfDNA *ESR1* mutations, Y537S/D538G. Interestingly, a total of 10 (83.3%) out of 12 MBC patients with increasing *ESR1* mutations were not response to any treatments (Figure 3A). In this study we were able to analyze, cfDNA *ESR1* mutations in serial samples from tumor tissues of 14 out of the total of 42 MBC patients. Two out of these14 patients had *ESR1* mutations in tumor tissue, but did not show an increase in cfDNA *ESR1* mutations. In contrast, six out of the 14 patients did not have *ESR1* mutations in the tumor tissue, but did show increases in cfDNA *ESR1* mutations.

**Table 2:** Patient characteristics of 12 ER-positive metastatic breast cancer cases with increassing cell-free DNA *ESR1* mutations

Case	Age at first blood sampling	Primary ER/PgR/ HER2/Ki67	Primary clinical stage	Metastatic ER/PgR/ HER2/Ki67	Site of tissue biopsy	Increasing cfDNA *ESR1* mutation			Treatment	Treatment after increasing cfDNA *ESR1* mutation	BOR to treatment after increasing cfDNA *ESR1* mutation
						2^nd^ blood draw	3^rd^ blood draw	4^th^ blood draw			
18	66	unknown/	I	90/60/-/6	Lung	Y537S	No elevating *ESR1* mutations	No elevating *ESR1* mutations	Fadrozole, chemotherapy, Letrozole, Toremifene, Anastrozole, MPA, Nab-Paclitaxel, Exemestane, Epirubicine+Cyclophosphamide,	Letrozole	PD
27	31	70/5/-/5	IIA	70/60/-/5	Lymph node	Y537S	-	-	FEC→ docetaxel, Zoladex, Zoladex+Tamoxifen, Zoladex + Anastrozole	Zoladex + Anastrozole	PD
44	58	-	IV	90/0/-/5	Breast	D538G	No elevating *ESR1* mutations	-	Anthracyclin+Cyclophosphamide, Trastuzumab, Letrozole, HdTOR+Trastuzumab, Exemestane+Trastuzumab, Nab-Paclitaxel+Trastuzumab, Letrozole, EE2, Fulvestrant	Fulvestrant	PD
53	40	-	IV	90/20/-/1	Ovary	Y537S	-	-	Exemestane, Pclitaxel, Letrozole, HdTOR, Capecitabine, Anastrozole, Docetaxel, Vinorelbine, Mitoxantrone+Mitomycin C+Methotrexate, Gemcitabine	Mitoxantrone Mitomycin C Methotrexate	PD
58	60	-	IV	90/90/-/10	Bone	No elevating *ESR1* mutations	Y537S	No elevating *ESR1* mutations	Letrozole+Zoladex, Tamoxifen, Exemestane, Adriamycin+Cyclophosphamide, Docetaxel, Paclitaxel, Mitoxantrone+Mitomycin C +Methotrexate	Paclitaxel	PD
62	67	60/5/-/10	IIB	5/0/-/30	Lymph node	Y537S	-	-	Docetaxel+Cyclophosphamide, Letrozole×4 years, Fulvestrant, Bevacizumab+Paclitaxel, Tamoxifen	Fulvestrant	PD
72	61	80/60/-/10	IIIA	90/80/-/2	Skin	Y537N	-	-	FEC, Anastrozole, Exemestane, HdTOR, Letrozole, EE2	EE2	PR
75	61	90/60/-	IIIA	90/0/-/20	Skin	No elevating *ESR1* mutations	Y537N	Y537N	CMF, Docetaxel, S-1+Anastrozple, Exemestane, Epirubicine+Cyclophosphamide, Docetaxel, Letrozole, EE2, Letrozole, EE2, Letrozole, Fulvestrant, Exemestane+everolimus	Fulvestrant/ Exemestane +everolimus	PD/SD
77	48	-	IV	10/0/1+/3	Bone	No elevating *ESR1* mutations	Y537S/D538G	-	CEF→Docetaxel, Exemestane, Fulvestrant, Zoladex+Anastrozole, HdTOR, MPA, Capecitabine+Cyclophosphamide, Letrozole, Vinolrelbine, HdTOR, Paclitaxel, Docetaxel	Docetaxel	PD
89	58	unknown	I	90/0/-/10	Breast	No elevating *ESR1* mutations	Y537N		Zoladex+Tamoxifen, MitomycineC+Fluorouracil +Epirubicine, Docetaxel, Zoladex+Tamoxifen, Zoladex+Anastrozole, Zoladex+Letrozole, S-1, Vinoreline, Nab-Paclitaxel, Exemestane, EE2, Letroozole, Fulvestrant	Fulvestrant	PD
101	68	70/0/-/15	I	100/10/-/30	Lung	Y537N	-	-	Letrozole, Toremifene, HdTOR,	HdTOR	SD
118	56	-/+/−/	IIA	90/80/-/5	Lymph node	Y537N	No elevating *ESR1* mutations	-	Adriamycine+Cyclophosphamide, Anastrozole, Exemestane, Letrozole, Docetaxel, Capecitabine, Letrozole, Exemestane, EE2, Letrozole, EE2	EE2	PR

Abbreviations: ER, estrogen receptor; PgR, progesteron receptor; HER2, human epidermal growth factor receptor 2; BOR, best overall response; FEC, Fluorourasil+epirubicine+cyclophosphamide; HdTOR, high dose toremifene; EE2, ethinylestradiol; CEF, cyclophosphamide+epirubicine+fluorouracil; MPA, medroxyprogesterone acetate; S-1, an oral fluoropyrimidine; PR, partial response; SD, stable disease; PD, progressive disease

### Survival analysis of the 42 MBC patients

Patients were grouped according to whether or not cfDNA *ESR1* mutations increased, and groups were compared by the patient response end points of time-to-treatment failure (TTF) and breast cancer-specific survival (BCSS) (Figure 3B, 3C). In the analysis of TTF, local recurrences, distant metastases, and disease progression at any site following blood sampling were considered as an event. In the analysis of BCSS, a total of 17 patients died of breast cancer, and these deaths were considered events. These were tested by Kaplan-Meier analysis and verified by the log-rank test. In TTF analysis, patients with no increase in cfDNA *ESR1* mutations had a longer time to failure than patients with increasing cfDNA *ESR1* mutations and these differences were significant by log-rank test (*P* = 0.0033). A total of 3 out of 12 patients with increasing cfDNA *ESR1* mutations were treated with chemotherapy. In BCSS analysis, there was no significant difference among the patients with and without increasing cfDNA *ESR1* mutations (*P* = 0.22).

### Representative clinical courses

**Figure 4:**
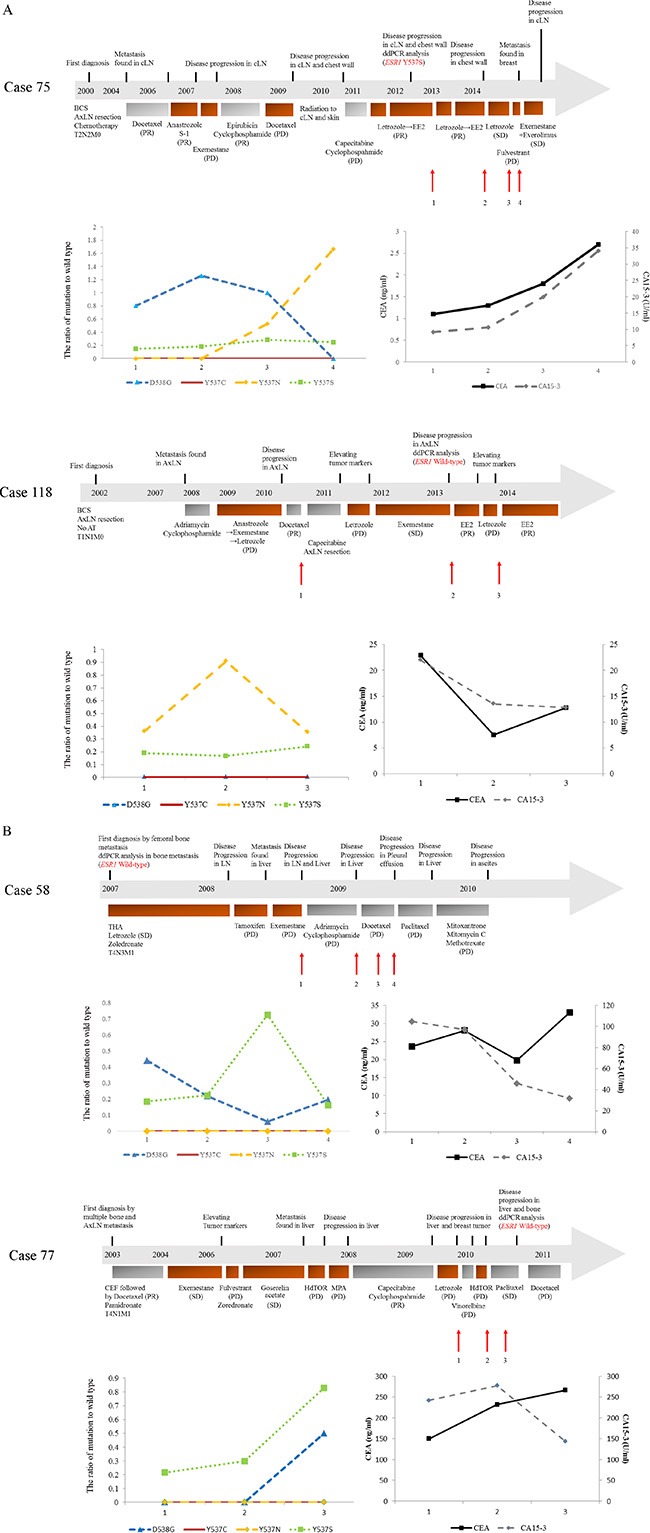
(**A, B**) show the clinical timelines for ER-positive MBC patients with increasing cfDNA *ESR1* mutations quantified using ddPCR. Patients’ histories of clinical treatment from first diagnosis are shown in the upper part. Each bar represents the timeline of treatment. Plasma levels of each cfDNA *ESR1* mutation are shown in the lower left and levels of tumor markers are shown in the lower right. During the tracking cfDNA *ESR1* mutations ratio, case 75 and case 118 were treated with endocrine therapy (A) and case 58 and case 77 were treated with chemotherapy (B). (A) Case 75 had increasing cfDNA *ESR1* Y537N ratio and decreasing cfDNA *ESR1* D538G during treatment and Case 118 had increasing cfDNA *ESR1* mutations in the first blood draw, but not in the third blood draw. (B) Case 58 and case 77 both showed increases in cfDNA *ESR1* mutations not in the second blood draw, but in the third blood draw. The existence of *ESR1* mutations in MBC tissue samples were analyzed previously [21]. Abbreviations; cLN, cervical lymph node; ddPCR, droplet digital polymerase chain reaction; BCS, breast conserving surgery; AxLN, axial lymph node; S-1, an oral fluoropyrimidine; PR, partial response; PD, progressive disease; SD, stable disease; EE2, ethinyl estradiol, THA, total hip arthroplasty; CEF, cyclophosphamide, epirubicin, and fluorouracil.

Four representative examples of changes in cfDNA *ESR1* mutations during the clinical course are highlighted below. During the tracking cfDNA *ESR1* mutations ratios, case 75 and case 118 were treated with endocrine therapy and case 58 and case 77 were treated with chemotherapy (Figure 4A, 4B). The existence of *ESR1* mutations in MBC tissue samples were analyzed previously [21].

In case 75, clinical metastasis was detected at 51 months after primary surgery. She then received systemic treatment with 9 different therapies including 5 endocrine therapies before the first blood draw. A metastatic chest wall tumor, which was biopsied before the first blood draw, showed *ESR1* Y537S beyond the selected cut-off level. Analysis of cfDNA identified *ESR1* mutations in the plasma sample; *ESR1* Y537N ratio was significantly increased in the third and the fourth blood draw in comparison with that in the first blood draw. Conversely, *ESR1* D538G ratio was significantly decreased in the fourth blood draw in comparison with that in the first blood draw. *ESR1* Y537S was slightly increased over the first blood draw and *ESR1* Y537C was not detected at any time-point. In the same blood samples, CEA and CA15-3 were gradually increased over the first blood draw. Hormonal therapy was effective at first, but she was not response to any endocrine therapies after the second blood draw. In case 118, axillary lymph node metastasis was detected at 70 months after primary surgery. She then received 5 systemic therapies including 3 endocrine therapies before the first blood draw. Metastatic axillary lymph nodes, which were collected at the same time as the second blood draw, did not have any *ESR1* mutations. Analysis of cfDNA identified *ESR1* mutations in the plasma sample; *ESR1* Y537N ratio in the second blood draw was significantly increased in comparison with the first blood draw, but in the third blood draw was decreased to the level of the first blood draw. Both *ESR1* Y537S and D538G ratio remained at the same level throughout the treatment period and *ESR1* Y537C was not detected at any time-point. In the same blood samples, CEA and CA15-3 showed that they decreased during the treatment period. About the effect of endocrine therapy, she was not response to letrozole after the first blood draw, but she was response to exemestane following the second blood draw. Interestingly, ethinyl estradiol (EE2) had been effective for almost one year following the third blood draw (Figure 4A).

Case 58 was diagnosed as stage IV invasive ductal carcinoma and had received 3 endocrine therapies before the first blood draw, but he did not receive endocrine therapies after the first blood draw. A metastatic bone tumor, which was biopsied before the first blood draw, did not have any *ESR1* mutations. Analysis of cfDNA identified *ESR1* mutations in the plasma sample; *ESR1* mutations tended to decrease after the first blood draw, but cfDNA *ESR1* Y537S ratio in the third blood draw was significantly increased in comparison with that in the first blood draw. In the same blood samples, tumor markers tended to decrease after the first blood draw, but CEA in the fourth blood draw was slightly increased. Any chemotherapy was not effective after the first blood draw. Case 77 was diagnosed as stage IV invasive ductal carcinoma and had received 8 systemic therapies including 6 endocrine therapies before the first blood draw. Interestingly, a metastatic bone tumor, which was biopsied after the third blood draw, did not have any *ESR1* mutations. Analysis of cfDNA identified *ESR1* mutations in the plasma sample; *ESR1* Y537S and D538G ratio in the third blood draw were significantly increased in comparison with that in the first blood draw. Neither *ESR1* Y537N nor Y537C were detected at any time-point. In the same samples, CEA tended to increase after the first blood draw and CA15-3 was slightly decreased in the last blood draw. Any endocrine therapy was not effective after the first blood draw, but he was response to paclitaxel (Figure 4B).

## DISCUSSION

The aim of this retrospective study was to investigate changes in cfDNA *ESR1* recurrent mutations in codons 537 and 538 with treatment in PBC and MBC patients using next-generation digital PCR platforms with a high level of sensitivity and specificity. The subjects of this serial study were a total of 77 women with PBC and 42 women with MBC. In looking at the number of dots plotted in Figure S1, S2 and Table S1, it appears that the majority if not all of the cases had some quantified value for one or more *ESR1* mutations. In addition, some untreated patients with PBC had detectable *ESR1* mutations in their plasma, which were not consistent with what it is known from the literature: *ESR1* LBD mutations are extremely rare in the early setting and can be identified in a significant proportion of MBC patients that have been exposed to AIs. The biggest reason is that, as our past history of publications reported, we had to use exceptionally high cutoffs to call a mutation because we took each cutoff of positive droplets widely to reduce bias by the rater. To solve this problem, we revised them by comparing 2-dimensional amplitudes of the same patient and used the PBC samples as a training set to establish the minimum cutoff for determining increases in *ESR1* mutation fraction.

Our study generated several interesting results with potential therapeutic implications. Firstly, we demonstrated that blood can be a sensitive source for the detection of *ESR1* mutations using ddPCR as several other groups have reported [9–11, 15, 18]. Secondly, the ratio of cfDNA *ESR1* mutations appeared to change during treatment. Increases in cfDNA *ESR1* mutations were observed in 13 samples of cfDNA from 12 (28.6%) out of 42 MBC patients (Table 2 and Figures 2, 3A). Among these 12 patients, 6 patients (50%) each had increasing cfDNA *ESR1* Y537S and Y537N, and 2 patients (16.7%) had increasing cfDNA *ESR1* D538G. We found that case 18, case 44, and case 118 showed increases in cfDNA *ESR1* mutations ratio in the second blood draw, but not in the third blood draw. Conversely, case 58, case 77, and case 89 showed increases in cfDNA *ESR1* mutations ratio not in the second blood draw, but in the third blood draw. Sequential increases in cfDNA *ESR1* Y537N were observed in case 75 (Table 2 and Figure 4). This finding raises the possibility that cfDNA *ESR1* mutations fluctuate easily as a result of treatment, compared with tumor tissue. Thus, there is a need to develop non-invasive methods to quickly assess mutational profiles across multiple metastases from an individual patient. Third, our results may support the previous hypothesis that *ESR1* mutations may be selected for after progression on AI therapy [5–7, 18]. In our study, a total of 11 out of 12 MBC patients with increasing *ESR1* mutations had been exposed to AIs for a long period. Interestingly, case 27, who had not been treated with AIs, had increasing cfDNA *ESR1* Y537S and showed a poor response to treatment with anastrozole (Table 2). Fourth, we demonstrate that increasing cfDNA *ESR1* mutations may be a poor predictor of post-treatment outcome. Of note, the higher the rate of *ESR1* mutations following therapy, the poorer response to treatment was. cfDNA *ESR1* mutations showed dynamic changes across serial plasma samples in 13 cfDNA samples from a total of 12 out of 42 MBC patients (28.6%). A total of 10 (83.3%) out of 12 MBC patients with increasing *ESR1* mutations were not response to any treatments (Table 2 and Figure 3A). In survival analysis, increasing numbers of cfDNA *ESR1* mutations may predict a shorter duration of post endocrine therapy effectiveness (*P* = 0.0033), but was not associated with BCSS (Figure 3B, 3C). This results had the possibility that cases with an increase in total cfDNA (where most of the increase was assumed to be coming from an increasing tumor burden [24–26]) had worse survival, but we could not find a correlation between the quantity of cfDNA and the frequency of each *ESR1* mutation. Therefore, our data on *ESR1* mutation presented here was independent on tumor burdens. Finally, two cases with increases in cfDNA *ESR1* mutations responded to treatment as follows; case 72–2 and case 118–2 were response to EE2 (Table 2 and Figure 4). This result may suggest that treatments different from conventional endocrine therapy in mechanism affect endocrine-resistant breast cancer [27, 28]. The number of PBC and MBC cases positive for each mutation should be presented clearly, but this was not provided in this study. The best way to provide the number of PBC and MBC cases positive for each mutation is compared the results of ddPCR with that of NGS, but we were not able to extract enough DNA to use for analysis of NGS. Even if we set cutoff using synthetic templates, it was challenging because there was a difference in distribution of droplets between synthetic templates and clinical samples. Additionally, in many articles that analyzed clinical samples using ddPCR, it seems to be difficult to judge positive droplets in 2-dimensional amplitude. Therefore, we mainly analyzed how each *ESR1* mutation changed by treatment in the same people.

The present study has limitations. This was a retrospective, single-institute study, and was prone to selection bias. Although the correlation of *ESR1* mutations between tumor tissue and plasma is closely associated with medical history of endocrine therapy, this studied population is heterogeneously treated and we had insufficient data to examine whether *ESR1* mutation detection is dependent on specific hormone therapies or not. The samples used in this study were obtained for biobanking. Therefore, a time from blood draw to spinning, freezing plasma and then thawing may affect the variability of the data.

In conclusion, our study demonstrates that ddPCR monitoring of recurrent *ESR1* mutations in cfDNA of breast cancer patients is feasible and is a useful method of providing relevant predictive information.

## MATERIALS AND METHODS

### Patients and breast cancer tissues

A total of 119 patients (253 plasma samples) with breast carcinoma, treated at Kumamoto University Hospital between 2004 and 2014, were enrolled in this study. Cases were selected if archival plasma samples were available from PBC before and after treatment and from MBC gathered more than twice at the time of progression. Informed consent was obtained from all patients before biopsy or surgery. The Ethics Committee of Kumamoto University Graduate School of Medicine (Kumamoto, Japan) approved the study protocol. Adjuvant and neoadjuvant treatment was administered in accordance with the recommendations of the St. Gallen international expert consensus on the primary therapy of early breast cancer [29–31]. The treatment of MBC patients was performed in accordance with the National Comprehensive Cancer Network Clinical Practice Guidelines in Oncology [32]. Patients were examined at the Kumamoto University Hospital or affiliated hospitals every 3 months for 5 years and every year thereafter. Recurrence was defined as the identification of positive spots by physical examination and/or by imaging diagnosis during the follow-up period. Metastatic patients were assessed monthly for clinical response, which was defined according to the Response Evaluation Criteria in Solid Tumors as complete response (CR), partial response (PR), stable disease (SD), or progressive disease (PD). We defined the presence of CR, PR, and long SD as responder and all other clinical responses as non- responder.

### Sample preparation

Blood collected in EDTA K2 tubes was processed as soon as possible and was centrifuged at 3000 rpm for 10 min, with plasma stored in freezers until DNA extraction. DNA was extracted from 200 μl of aliquots of plasma using the PureLink^®^ Viral RNA/DNA Mini Kit (ThermoFisher scientific, Waltham, MA USA) according to the manufacturer's instructions. All DNA extracts were quantified using a NanoDrop 2000 spectrometer (NanoDrop Technologies, Wilmington, DE, USA) and purity was determined from the A260/A280 absorbance ratios.

### Analysis of *ESR1* mutations by ddPCR

The ddPCR assay for the detection of the variant types of amino acids 537 and 538 in *ESR1* exon 8 consisted of a pair of primers and two *Taq* Man minor groove binding probes, as described previously, and this assay was carried out in the same sample twice using the QX200^™^ Droplet Digital^™^ PCR System (Bio-Rad laboratories, Hercules, CA, USA) as described previously [21]. The PCR data were quantified as copies/μl using QuantaSoft^™^ software (Bio-Rad laboratories). The ddPCR method had been optimized by comparative analysis of a dilution series of synthetic copies of each indicated mutant *ESR1* oligonucleotide, as reported previously [21]. In addition, to be satisfied that the 4 assays have sufficiently good enough performance, we performed comparative analysis of the dilution series and cross-reactivity of indicated synthetic each *ESR1* mutation oligonucleotides of *ESR1* Y537S, Y537N, Y537C, and D538G, in a background of wild-type normal human DNA (Figure S3). The dilution experiments were prepared by two-fold serial dilution of each synthetic *ESR1* mutation stock oligonucleotide in a background of wild-type normal human DNA (TaqMan Control Genomic DNA) where the total DNA content of each ddPCR reaction was 20 ng and “wild-type double” was 40 ng. The experiments for cross-reactivity between mutations were prepared by 5% each synthetic *ESR1* mutation stock oligonucleotide in a background of wild-type normal human DNA where the total DNA content of each ddPCR reaction was 20 ng. We confirmed that this assay was able to detect each *ESR1* mutant molecule in a background of wild-type normal human DNA with the lowest concentration and was not able to detect any false-positives in the wild-type normal human DNA.

### Probes and primers

Immunohistochemical staining for estrogen receptor alpha (ERα), progesterone receptor (PgR), and human epidermal growth factor receptor 2 (HER2) staining, and Ki67 and the evaluation of them was described previously [33].

### Statistical analysis

The nonparametric Mann-Whitney *U* test and contingency analysis were adopted for statistical analysis of the associations between cfDNA *ESR1* mutations ratio and clinicopathological factors, and between the differences with cfDNA *ESR1* mutations ratio in the first blood sample. For TTF and BCSS, Kaplan–Meier method was used to estimate survival rates, and differences between survival curves were evaluated by the log-rank test. Differences were considered significant when a *P*-value < 0.05 was obtained. All statistical analyses were two-sided and were performed using JMP software version 10.0.1 for Windows (SAS institute Japan, Tokyo, Japan).

## SUPPLEMENTARY FIGURES AND TABLES




