# Supplementary material for: Clinical significance of monitoring ESR1 mutations in circulating cell-free DNA in estrogen receptor positive breast cancer patients
Source: Oncotarget. 2016 Apr 19;7(22):32504–18. doi: 10.18632/oncotarget.8839 (PMC5078029; doi:10.18632/oncotarget.8839)
Supplement: Supplementary file 1 [file oncotarget-07-32504-s001.pdf]

Clinical significance of monitoring *ESR1* mutations in circulating cell-free DNA in estrogen receptor positive breast cancer patients

Supplementary Materials

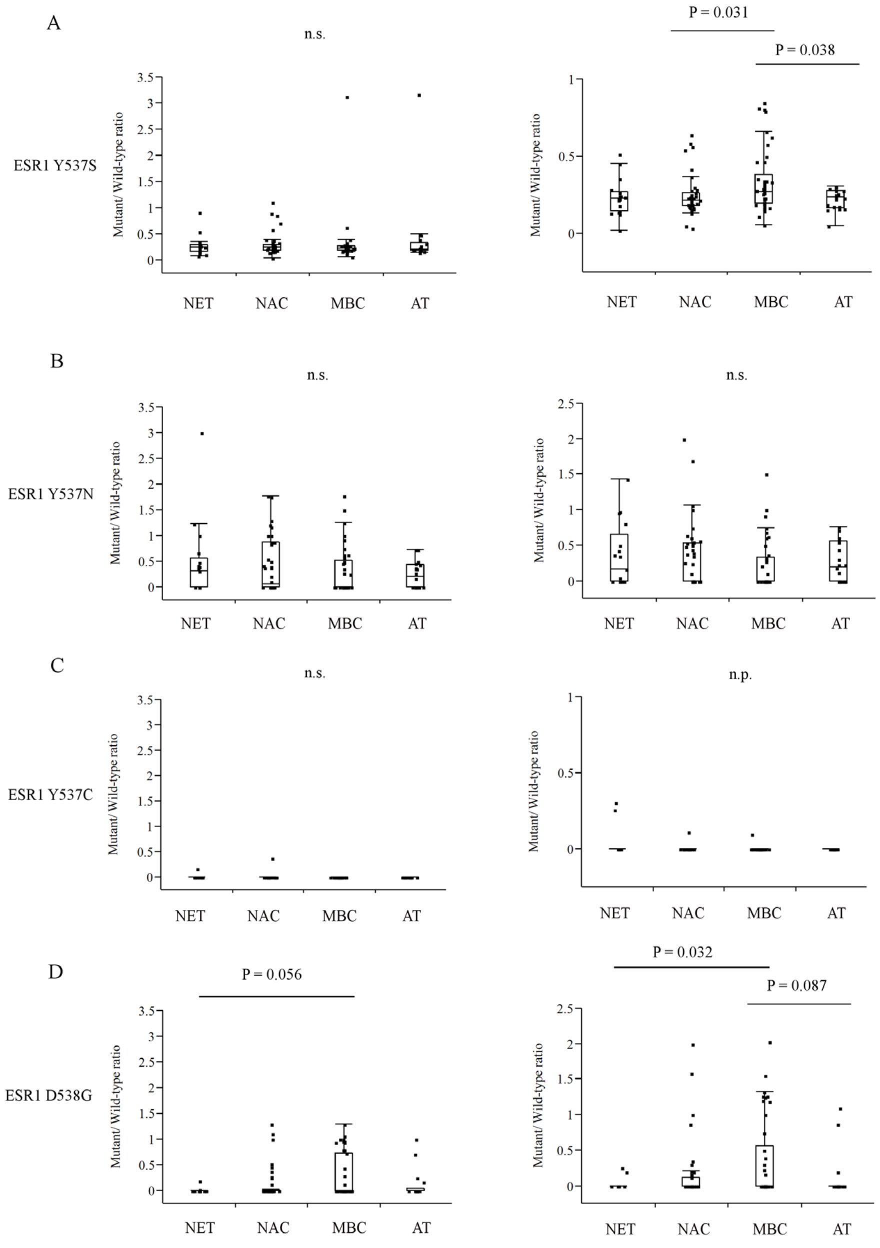

Supplementary Figure S1: (**A–D**) The ratio of mutation to wild type for the 4 cfDNA *ESR1* mutations (A: *ESR1* Y537S, B: Y537N, C: Y537C, and D: D538G) for the 1st (left side) and the 2nd blood draw (right side) in this study are shown. In each figure, the NET group, the NAC group, the MBC group, and the AT group from the left to the right is shown. In the 1st blood draw, there was no significant difference between the PBC group and the MBC group. However, in the 2nd blood draw, cfDNA *ESR1* Y537S ratio in the MBC group was significantly higher than that in the NAC and the AT group (P = 0.031 and 0.038, respectively), and cfDNA *ESR1* D538G ratio was significantly higher than that in the NET group (P = 0.032). Abbreviations; cfDNA, cell-free DNA; NET, neo-adjuvant therapy; NAC, neo-adjuvant chemotherapy; MBC, metastatic breast cancer; AT, adjuvant therapy; PBC, primary breast cancer; n.s., not significant.

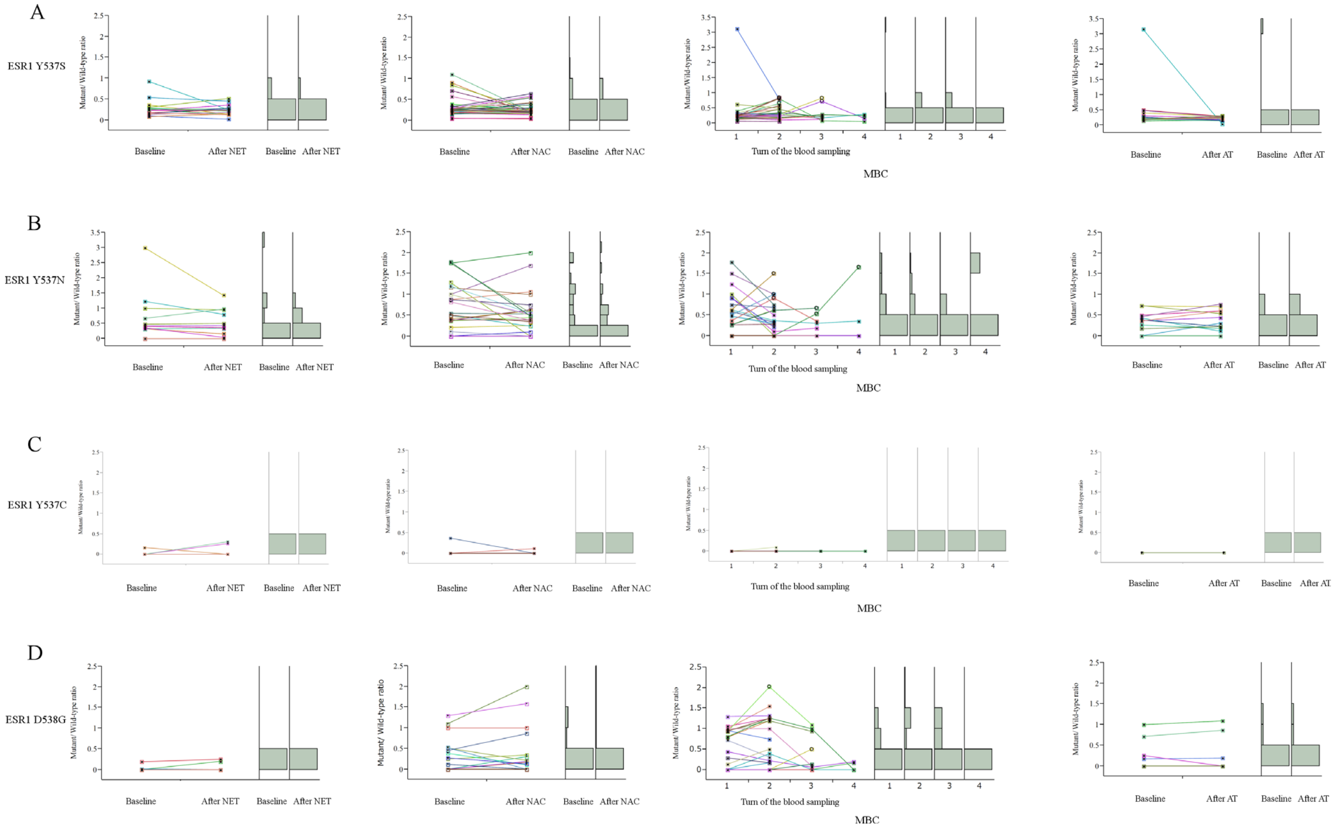

Supplementary Figure S2: (**A–D**) show the actual changes and their histograms in each cfDNA *ESR1* mutation ratio (A: *ESR1* Y537S, B: Y537N, C: Y537C, and D: D538G) during the treatment in PBC groups and the MBC group. In each figure, the NET group, the NAC group, the MBC group, and the AT group from the left to the right is shown. Abbreviations; cfDNA, cell-free DNA; PBC, primary breast cancer; MBC, metastatic breast cancer; NET, neo-adjuvant therapy; NAC, neo-adjuvant chemotherapy; AT, adjuvant therapy.

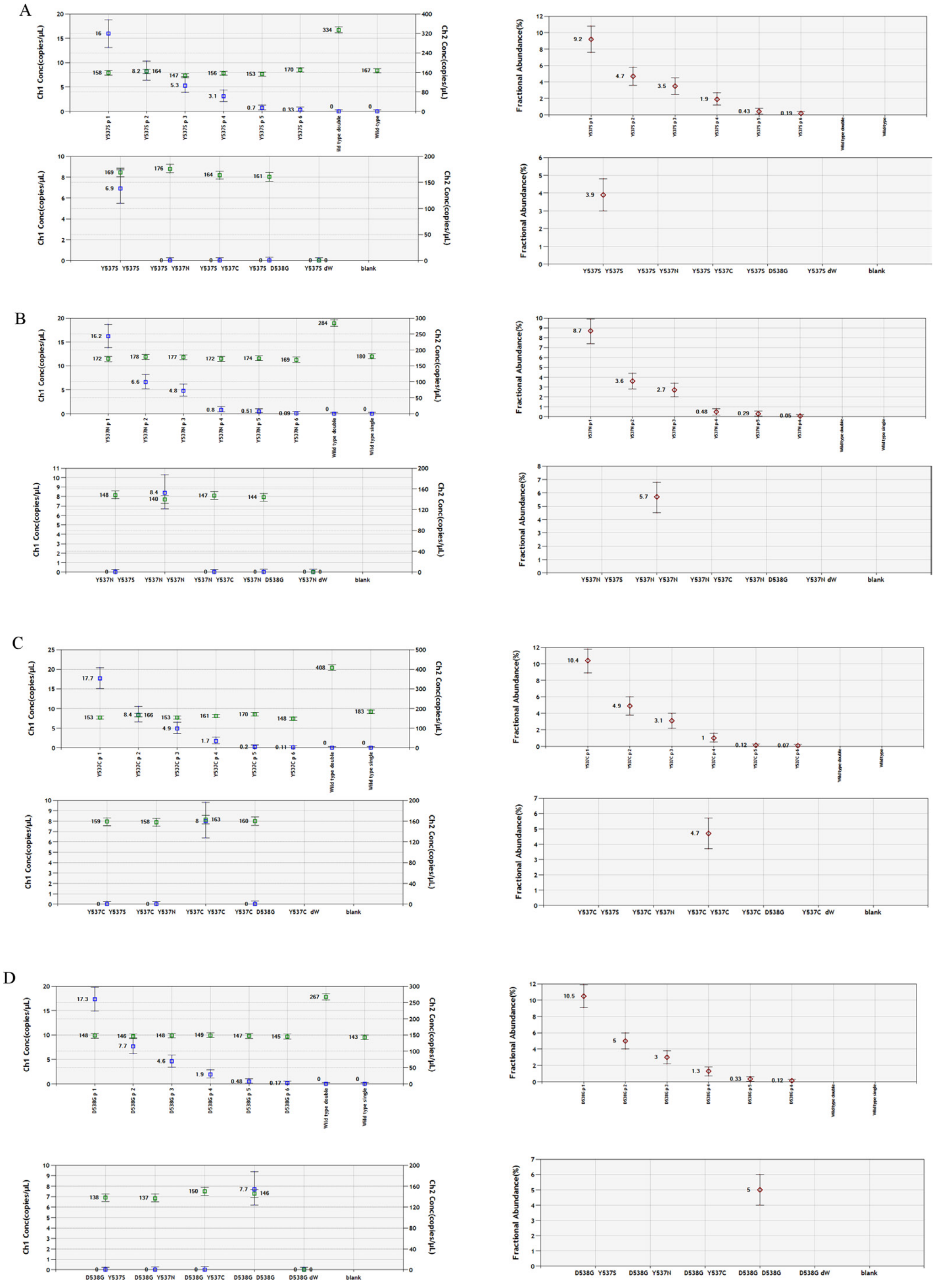

Supplementary Figure S3: (**A–D**) Comparative analysis of the dilution series (upper part) and cross-reactivity (lower part) of indicated synthetic each *ESR1* mutation oligonucleotides in a background of wild-type normal human DNA using the detection probe of A; *ESR1* Y537S, B; Y537N, C; Y537C, and D; D538G are present. Left panel shows the absolute copies/µl of indicated synthetic each *ESR1* mutation oligonucleotides and wild-type normal human DNA and right panel shows corresponding fractional abundance (%). The dilution experiments were prepared by two-fold serial dilution of each synthetic *ESR1* mutation stock oligonucleotide in a background of wild-type normal human DNA (TaqMan Control Genomic DNA) where the total DNA content of each ddPCR reaction was 20 ng and "wild-type double" was 40 ng. The experiments for cross-reactivity between mutations were prepared by 5% each synthetic *ESR1* mutation stock oligonucleotide in a background of wild-type normal human DNA where the total DNA content of each ddPCR reaction was 20 ng. We confirmed that this assay was able to detect each *ESR1* mutant molecule in a background of wild-type normal human DNA with the lowest concentration and was not able to detect any false-positives in the wild-type normal human DNA. Abbreviation; dW, distilled water.

Supplementary Table S1: Association of each *ESR1* mutation ratio with clinicopathological parameters in primary and metastatic breast cancer patients at first blood sampling. See Supplementary_Table_S1
