# Supplementary material for: Clinical significance of monitoring ESR1 mutations in circulating cell-free DNA in estrogen receptor positive breast cancer patients
Source: Oncotarget. 2016 Apr 19;7(22):32504–18. doi: 10.18632/oncotarget.8839 (PMC5078029; doi:10.18632/oncotarget.8839)
Supplement: Supplementary file 2 [file oncotarget-07-32504-s002.docx]

Supplementary Table S1: Association of each ***ESR1*** mutation ratio with clinicopathological parameters in primary and metastatic breast cancer patients at first blood sampling

|  |  |  |  | **Primary breast cancer** | | | |  |  |  |  |  | **M etastatic breast cancer** | | | |  |  |
| --- | --- | --- | --- | --- | --- | --- | --- | --- | --- | --- | --- | --- | --- | --- | --- | --- | --- | --- |
| **Variables** | **Total** |  |  | ***ESR1* genomic state, median ratio  (25%, 75%)** | | | |  |  | **Total** |  |  | ***ESR1* genomic state, median ratio  (25%, 75%)** | | | |  |  |
|  | (*N* = 77) | Y537S | *P* value | Y537N | *P* value | Y537C | *P* value | D538G | *P* value | (N = 42 ) | Y537S | *P* value | Y537N | *P* value | Y537C | *P* value | D538G | *P* value |
| **Age at biopsy** |  |  |  |  |  |  |  |  |  |  |  |  |  |  |  |  |  |  |
| < 50 | 26 | 0.25  (0.18, 0.33) | 0.67 | 0.20 (0, 1) | 0.47 | 0 (0, 0) | 0.61 | 0 (0, 0) | 0.95 | 15 | 0.23  (0.22, 0.26) | 0.26 | 0 (0, 0.92) | 0.58 | 0 (0, 0) | 1 | 0 (0, 0) | 0.35 |
| > 50 | 51 | 0.24  (0.18, 0.29) |  | 0.21 (0, 0.48) |  | 0 (0, 0) |  | 0 (0, 0) |  | 27 | 0.21 (0.17, 0.31) |  | 0 (0, 0.46) |  | 0 (0, 0) |  | 0 (0, 0.8) |  |
| **Primary clinical stage** | |  |  |  |  |  |  |  |  |  |  |  |  |  |  |  |  |  |
| I | 19 | 0.22  (0.18, 0.30) | 0.57 | 0.36 (0, 0.57) | 0.27 | 0 (0, 0) | 0.47 | 0 (0, 0) | 0.44 | 7 | 0.23  (0.17, 0.25) | 0.76 | 0.26 (0, 1) | 0.039* | 0 (0, 0) | 1 | 0 ( 0, 0) | 0.13 |
| II | 44 | 0.25 (0.19, 0.33) |  | 0 (0, 0.49) |  | 0 (0, 0) |  | 0 (0, 0) |  | 15 | 0.24  (0.18, 0.31) |  | 0 (0, 0.26) |  | 0 (0, 0) |  | 0 (0, 0.8) |  |
| III | 14 | 0.25  (0.17, 0.28) |  | 0.52 (0, 1.2) |  | 0 (0, 0) |  | 0 (0, 0) |  | 3 | 0.27  (0.20, 0.34) |  | 0.75 (0.5, 1.8) |  | 0 (0, 0) |  | 0 (0, 0) |  |
| IV | 0 |  |  |  |  |  |  |  |  | 17 | 0.22  (0.18, 0.26) |  | 0 (0, 0.46) |  | 0 (0, 0) |  | 0 (0, 0.94) |  |
| **Histological type** | |  |  |  |  |  |  |  |  |  |  |  |  |  |  |  |  |  |
| Invasive ductal | 73 | 0.24  (0.19, 0.31) | 0.32 | 0.21 (0, 0.7) | 0.61 | 0 (0, 0) | 0.94 | 0 (0, 0) | 0.8 | 40 | 0.22  (0.18, 0.28) | 0.55 | 0 (0, 0.47) | 0.084 | 0 (0, 0) | 1 | 0 (0, 0.77) | 0.35 |
| Invasive lobular | 1 | 0.16 |  | 0 |  | 0 (0, 0) |  | 0 (0, 0) |  | 2 | 0.24  (0.23, 0.25) |  | 0.625 |  | 0 (0, 0) |  | 0 (0, 0) |  |
| M ucinous | 3 | 0.23 (0.18, 0.24) |  | 0.36 (0, 0.41) |  | 0 (0, 0) |  | 0 (0, 0.19) |  | 0 |  |  |  |  |  |  |  |  |
| **Histological grade** | |  |  |  |  |  |  |  |  |  |  |  |  |  |  |  |  |  |
| 1 | 27 | 0.26 (0.19, 0.34) | 0.43 | 0.17 (0, 0.43) | 0.61 | 0 (0, 0) | 0.57 | 0 (0, 0.016) | 0.78 | 18 | 0.21 (0.18, 0.25) | 0.63 | 0 (0, 0.40) | 0.24 | 0 (0, 0) | 1 | 0 (0, 0.072) | 0.32 |
| 2 | 39 | 0.24 (0.17, 0.28) |  | 0.32 (0, 0.82) |  | 0 (0, 0) |  | 0 (0, 0) |  | 12 | 0.27  (0.22, 0.29) |  | 0 (0, 0.68) |  | 0 (0, 0) |  | 0 (0, 0.99) |  |
| 3 | 10 | 0.25  (0.18, 0.35) |  | 0.18 (0, 1.2) |  | 0 (0, 0) |  | 0 (0, 0.063) |  | 10 | 0.20 (0.14, 0.31) |  | 0 (0, 0.15) |  | 0 (0, 0) |  | 0 (0, 0.79) |  |
| Lobular | 1 | 0.16 |  | 0 |  | 0 (0, 0) |  | 0 (0, 0) |  | 2 | 0.24 (0.23, 0.25) |  | 0.625 |  | 0 (0, 0) |  | 0 (0, 0) |  |
| **Percentage of ERa immunostaining** | | |  |  |  |  |  |  |  |  |  |  |  |  |  |  |  |  |
| < 80 | 30 | 0.24  (0.18, 0.30) | 0.94 | 0 (0, 0.58) | 0.18 | 0 (0, 0) | 0.26 | 0 (0, 0) | 0.86 | 20 | 0.24  (0.18, 0.27) | 0.83 | 0 (0, 0.49) | 0.83 | 0 (0, 0) | 1 | 0 (0, 0.22) | 0.58 |
| > 80 | 47 | 0.24 (0.18, 0.30) |  | 0.38 (0, 0.67) |  | 0 (0, 0) |  | 0 (0, 0) |  | 22 | 0.22  (0.19, 0.30) |  | 0 (0, 0.59) |  | 0 (0, 0) |  | 0 (0, 0.79) |  |
| PgR |  |  |  |  |  |  |  |  |  |  |  |  |  |  |  |  |  |  |
| Negative | 9 | 0.25  (0.22, 0.28) | 0.56 | 0.36 (0, 0.85) | 0.52 | 0 (0, 0) | 0.61 | 0 (0, 0) | 0.6 | 9 | 0.25  (0.21, 0.30) | 0.24 | 0 (0, 0.31) | 0.37 | 0 (0, 0) | 1 | 0 (0, 0.82) | 0.59 |
| Poseitive | 68 | 0.24  (0.18, 0.31) |  | 0.14 (0, 0.56) |  | 0 (0, 0) |  | 0 (0, 0) |  | 33 | 0.22  (0.18, 0.26) |  | 0 (0, 0.54) |  | 0 (0, 0) |  | 0 (0, 0.62) |  |
| HER2 |  |  |  |  |  |  |  |  |  |  |  |  |  |  |  |  |  |  |
| Negative | 67 | 0.24  (0.18, 0.29) | 0.42 | 0.17 (0, 0.5) | 0.35 | 0 (0, 0) | 0.58 | 0 (0, 0) | 0.1 | 36 | 0.23  (0.20, 0.28) | 0.052 | 0 (0, 0.59) | 0.21 | 0 (0, 0) | 1 | 0 (0, 0.65) | 0.74 |
| Positive | 10 | 0.26  (0.21, 0.44) |  | 0.59 (0, 1) |  | 0 (0, 0) |  | 0 (0, 0) |  | 6 | 0.18 (0.11, 0.25) |  | 0 (0, 0.15) |  | 0 (0, 0) |  | 0 (0, 0.96) |  |
| Ki67 LI |  |  |  |  |  |  |  |  |  |  |  |  |  |  |  |  |  |  |
| < 14 | 33 | 0.24  (0.17, 0.28) | 0.93 | 0.36 (0, 0.52) | 0.75 | 0 (0, 0) | 0.78 | 0 (0, 0) | 0.94 | 21 | 0.22 (0.19, 0.26) | 0.76 | 0.26 (0, 0.56) | 0.19 | 0 (0, 0) | 1 | 0 (0, 0) | 0.048* |
| > 14 | 23 | 0.24  (0.18, 0.27) |  | 0 (0, 0.88) |  | 0 (0, 0) |  | 0 (0, 0) |  | 12 | 0.24 (0.18, 0.28) |  | 0 (0, 0.44) |  | 0 (0, 0) |  | 0.40 (0, 0.99) |  |
| Unknown | 21 |  |  |  |  |  |  |  |  | 9 |  |  |  |  |  |  |  |  |
| **Therapy change before biopsy** | | |  |  |  |  |  |  |  |  |  |  |  |  |  |  |  |  |
| 0 |  |  |  |  |  |  |  |  |  | 14 | 0.22 (0.20, 0.24) | 0.066 | 0 (0, 0.5) | 0.56 | 0 (0, 0) | 1 | 0 (0, 0072) | 0.6 |
| 1, 2 |  |  |  |  |  |  |  |  |  | 15 | 0.27 (0.20, 0.34) |  | 0 (0, 0.75) |  | 0 (0, 0) |  | 0 (0, 0.72) |  |
| 3 < |  |  |  |  |  |  |  |  |  | 13 | 0.21 (0.16, 0.25) |  | 0 (0, 0.31) |  | 0 (0, 0) |  | 0 (0, 0.93) |  |

Abbreviations: ERa, estrogen receptor alpha; PgR, progesteron receptor; HER2, human epidermal growth factor receptor 2, LI, labeling index.
